# *Cellulosimicrobium* Infections in Humans—A Narrative Review

**DOI:** 10.3390/antibiotics13060562

**Published:** 2024-06-16

**Authors:** Petros Ioannou, Alexandra Vorria, George Samonis

**Affiliations:** 1 School of Medicine, University of Crete, 71003 Heraklion, Greece; 2 University Hospital of Heraklion, 71110 Heraklion, Greece; 3 Metropolitan Hospital, Neon Faliron, 18547 Athens, Greece

**Keywords:** bacteremia, infective endocarditis, peritoneal dialysis, peritonitis, *Cellulosimicrobium*, *Oerskovia*

## Abstract

*Cellulosimicrobium* species (formerly known as *Oerskovia*) are Gram-positive filamentous bacteria in the family Promicromonosporaceae and are more commonly found in sewage and soil. The present study aimed to identify all the published cases of *Cellulosimicrobium* species infections in the literature, describe the epidemiological, clinical, and microbiological characteristics, and provide data regarding its antimicrobial resistance, treatment, and outcomes. A narrative review was performed based on a PubMed and Scopus database search. In total, 38 studies provided data on 40 patients with infections by these species. The median age of patients was 52.5 years, and 55% were male. The most common infection types were bacteremia, infective endocarditis (IE), osteoarticular infections, peritoneal dialysis-associated peritonitis, and endophthalmitis. Antimicrobial resistance to vancomycin and the combination of trimethoprim and sulfamethoxazole was minimal, and vancomycin was the most commonly used antimicrobial for treating these infections. Overall mortality was minimal for all infections, except for bacteremia and IE, which carried high mortality rates.

## 1. Introduction

Novel microbiological diagnostic tools occupying modern genetic and molecular methods have recently been introduced into the practice of microbiological laboratories in large hospitals. These tools include 16s rRNA and matrix-assisted laser desorption/ionization time-of-flight mass spectrometry (MALDI-TOF MS). These new diagnostic modalities have led to the accurate and more frequent identification of relatively rare microorganisms [1,2]. Such microorganisms may have been very difficult identify with classical microbiological tools that assess the morphology and biochemical profile of these microorganisms [3].

*Cellulosimicrobium* species (formerly known as *Oerskovia*) are Gram-positive filamentous bacteria in the family Promicromonosporaceae and are more commonly found in sewage and soil. The genus *Cellulosimicrobium* was proposed in 2001 by Schumann et al. and replaced the nomenclature of *Oerskovia* and other relevant bacteria, such as *Cellulomonas cellulans* [4]. They rarely cause infections in humans, with only a few dozen cases being described in the literature until now [5]. Most studies on this pathogen are case reports with brief literature reviews. These infections often occur in patients who are immunocompromised or have other underlying conditions [5]. Due to the rarity of this microorganism, its pathogenic potential, as well as the epidemiological, clinical, and microbiological characteristics associated with these infections, have not been adequately described.

The present study aimed to identify all cases of *Cellulosimicrobium* species infections published in the literature and provide data about epidemiological, clinical, and microbiological characteristics, as well as data regarding antimicrobial resistance, treatment, and outcomes.

## 2. Materials and Methods

The methodology of the present review included screening the literature to identify studies that provided original information on human infections caused by *Cellulosimicrobium* species. Two investigators (A.V. and P.I.) independently searched PubMed and Scopus databases for eligible articles reporting “(*Cellulosimicrobium* OR *Oerskovia*) AND infection” until 24 February 2024. Any differences between the two investigators were solved by consensus. All data regarding infections from case reports and case series providing information at least about epidemiology, microbiology, treatment, and outcomes of *Cellulosimicrobium* species infections in humans were included. Reviews, either narrative or systematic, letters to the editor, and other non-original studies were excluded. Only studies in the English language were included, while those with no access to the full text, those presenting aggregated data, and those not referring to humans were also excluded. Additionally, studies without information on patients’ epidemiology and mortality were excluded. The references in the included articles were searched to identify other potentially relevant studies that may have been previously missed.

Two investigators (P.I. and A.V.) extracted all relevant information from the eligible studies. Data regarding age, epidemiology characteristics, infection site, microbiology, antimicrobial susceptibility, antimicrobial treatment, and outcomes of human *Cellulosimicrobium* species infections were extracted and further analyzed.

## 3. Results

### 3.1. Characteristics of Studies Included in the Review

**Figure 1:**
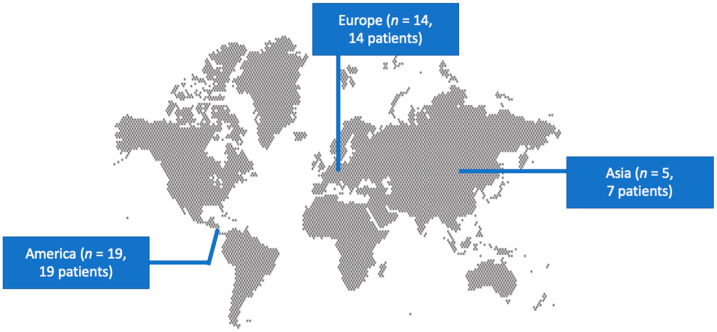
Geographical distribution of *Cellulosimicrobium* species infections worldwide.

The literature search yielded 133 studies. After screening all potentially eligible articles and assessing the references of the included articles, only 38 studies met the inclusion criteria and were considered for data extraction [5,6,7,8,9,10,11,12,13,14,15,16,17,18,19,20,21,22,23,24,25,26,27,28,29,30,31,32,33,34,35,36,37,38,39,40,41,42]. These 38 studies provided information on 40 patients. Among them, 19 studies were conducted in North and South America, 14 in Europe, and 5 in Asia. There were 37 case reports and 1 case series. Figure 1 shows a graphical representation of the geographical distribution of the published cases. Table 1 shows the characteristics of the included studies in the present review.

### 3.2. Epidemiology of Cellulosimicrobium Species Infections

The age of patients ranged from a few days to 82 years; the median was 52.5 years. Out of 40 patients, 22 (55%) were male. Regarding their history, 15% (6 patients) had surgery during the three months before the infection’s diagnosis. Among all patients with available data, 33.3% (13 out of 39) had recently received antimicrobial therapy, 27.5% (11 out of 40) had a central venous catheter (CVC), 15% (6) had a history of end-stage renal disease (ESRD), 12.5% (5) were on peritoneal dialysis (PD), 2.5% (1) were on hemodialysis, and 15% (6) had an active malignancy, with 7.5% (3) having a hematological malignancy. Most patients with malignancies (5 out of 6) were on chemotherapy. Additionally, 10% (4) had a prosthetic cardiac valve, and 2.5% (1) had a history of previous infective endocarditis (IE).

### 3.3. Microbiology and Antimicrobial Resistance of Cellulosimicrobium Species Infections

*Cellulosimicrobium* species were isolated from the blood in 55% (22 patients), peritoneal fluid in 12.5% (5), ocular fluid in 12.5% (5), synovial fluid or tissue in 5% (2), tissue biopsy in 5% (2), cerebrospinal fluid in 2.5% (1), deep wound samples in 2.5% (1), pus culture in 2.5% (1), valve culture in 2.5% (1, who also had positive blood cultures) and bile culture in 2.5% (1). Infection was polymicrobial in 12.5% (5 patients), with the other identified species being methicillin-resistant *Staphylococcus aureus*, coagulase-negative *Staphylococcus*, and *Escherichia coli* in one patient, *Comamonas acidovorans* in another one, *Myroides* species in a third, *Bacillus* species in a fourth, and *Enterobacter cloacae* in the last one. Identification was based on 16s rRNA sequencing in 40% (16 patients) and MALDI-TOF MS in 15% (6). The identification method was not reported in 57.5% (23 patients).

The identified species were *Cellulosimicrobium cellulans* or *Oerskovia xanthineolytica* (the older name of the same pathogen) in 67.5% (27 patients), *Oerskovia turbata* in 15% (6), and *Cellulosimicrobium funkei* in 5% (2), while the species were not reported in 12.5% (5).

The most commonly used method for susceptibility testing was dilution in 38.1% (8 out of 21 patients with available data), disk diffusion in 28.6% (6), disk diffusion and dilution in 14.3% (3), E-test in 14.3% (3), and paper diffusion in 4.8% (1). Resistance to clindamycin was 70% (7 out of 10 patients with available data), to penicillin was 60% (12 out of 20), to aminopenicillins was 53.3% (8 out of 15), to cephalosporins was 42.9% (9 out of 21 patients with available data), to aminoglycosides was 33.3% (6 out of 18), to tetracyclines was 29.4% (5 out of 17), to carbapenems was 16.7% (2 out of 12), to the combination of trimethoprim and sulfamethoxazole was 8.7% (2 out of 23), and to vancomycin was 0% (0 out of 29).

### 3.4. Clinical Presentation of Cellulosimicrobium Species Infections

The most common *Cellulosimicrobium* species infections were those of the bloodstream in 55% (22 out of 40 patients), IE in 20% (8), osteoarticular infections in 15% (6), peritonitis in 12.5% (5), endophthalmitis in 12.5% (5), central nervous system infections in 5% (2), lower respiratory tract infections in 5% (2), urinary tract infections (UTIs) in 2.5% (1), and skin and soft tissue infection (SSTI) in 2.5% (1). The most common clinical symptoms were fever in 52.8% (19 out of 36 patients) and sepsis in 28.9% (11 out of 38).

### 3.5. Treatment and Outcomes of Cellulosimicrobium Species Infections

The antimicrobial agents more commonly used were vancomycin in 68.4% (26 out of 38 patients), cephalosporins in 28.9% (11), aminoglycosides in 26.3% (10), the combination of trimethoprim with sulfamethoxazole in 21.1% (8), rifampicin in 15.8% (6), carbapenems in 13.2% (5), penicillin in 10.5% (4), aminopenicillins in 10.5% (4), linezolid in 10.5% (4), clindamycin in 7.9% (3), macrolides in 7.9% (3), and quinolones in 7.9% (3). The median treatment duration among survivors was 21.5 days, ranging from 7 to 264 days, with an interquartile range of 14 to 57.8 days. Overall mortality was 20% (8 out of 40 patients) and was attributed directly to the infection in 17.5% (7 patients). Table 2 shows the characteristics of patients with *Cellulosimicrobium* species infections in total and in regards to the type of infection.

### 3.6. Bacteremia Due to Cellulosimicrobium Species

Twenty-two patients had bacteremia caused by *Cellulosimicrobium* species. The median age of bacteremic patients was 52.5 years, ranging from a few days to 82 years, and had an interquartile range of 37.5 to 70.5 years; 50% (11 out of 22 patients) were male. Among all, 27.3% (6 patients) had an active malignancy (hematological in half of them), with 23.8% (5) being on chemotherapy and 19% (4) having neutropenia. Moreover, 50% (11 patients) had a CVC, 42.9% (9) had received antimicrobial therapy during the past three months, 18.2% (4) had a prosthetic cardiac valve, and 4.5% (1) had recent cardiac surgery. Among patients with bacteremia, 36.4% (8 patients) had IE, 9.1% (2) had osteoarticular infections, 4.6% (1) had a central nervous system infection, and 4.6% (1) had an SSTI. The infection was polymicrobial in 9.1% (2 patients), and the most common clinical characteristics were fever in 84.2% (16 out of 19 patients with available data), sepsis in 52.4% (11 out of 21), and shock in 19% (4). The median duration of treatment was 21 days. Mortality was 36.4% (8 out of 22 patients) and was directly attributed to the infection in 31.8% (7).

A comparison among patients with bacteremia who survived and those who died showed that patients who died were older, were more commonly female, and were more likely to have sepsis and a diagnosis of IE. However, the small number of patients in this analysis precluded the conduct of statistical analysis. Notably, no significant differences were noted among patients with immunosuppression among these two subgroups. Table 3 shows the characteristics of patients with bacteremia in regards to patients’ outcomes.

### 3.7. Infective Endocarditis Due to Cellulosimicrobium Species

Eight patients had IE caused by *Cellulosimicrobium* species. The median age of patients was 67 years, ranging from 30 to 82 years, with an interquartile range of 52.3 to 80.3; 37.5% (3 out of 8 patients) were male. Among all patients, 50% (4) had a prosthetic cardiac valve, 12.5% (1) had a CVC, 12.5% (1) had hematological malignancy on chemotherapy, 12.5% (1) had a history of IE, and 12.5% (1) had a history of recent cardiac surgery. Bacteremia was present in all patients with IE, while a concurrent osteoarticular infection was present in 12.5% (1 patient). In 62.5% (5 patients), the infected valve was the aortic, in 25% (2) the mitral, and in 12.5% (1) the tricuspid. The diagnosis was facilitated by transesophageal echocardiography in 62.5% (5 patients), transthoracic echocardiography in 12.5% (1), and autopsy, in 25% (2). Fever was present in 87.5% (7 patients), sepsis in 50% (4), and shock in 12.5% (1). Heart failure was diagnosed in 25% (2 patients), embolic phenomena occurred in 37.5% (3), paravalvular abscess in 25% (2), and immunological phenomena in 12.5% (1). The median duration of treatment was 63 days. Mortality was 62.5% and was directly attributed to the infection in all patients.

### 3.8. Osteoarticular Cellulosimicrobium Species Infections

Six patients had osteoarticular infections caused by *Cellulosimicrobium* species. Their median age was 61 years (ranging from 5 to 81), while the interquartile range was 14–78.8; 83.3% (5 out of 6 patients) were male. No patient had a predisposing factor, but 50% (3) had received antimicrobial therapy during the previous three months. Among these patients, 33.3% (2 patients) had bacteremia, 16.7% (1) had IE, and 16.7% (1) had an SSTI. Infection was polymicrobial in 16.7% (1). Among patients with available data, none had fever, but 16.7% (1 patient) had sepsis. The median duration of treatment was 45.5 days. No patient died.

### 3.9. Cellulosimicrobium Species Peritonitis

This review identified five patients with peritonitis due to Cellulosimicrobium species. Their median age was 59 years (ranging from 13 to 70); 40% (2 patients) were male. All patients had ESRD and were on PD. The diagnosis was based on the characteristics and culture of the peritoneal fluid. The infection was polymicrobial in 20% (1 patient). Fever was present in 50% (2 out of 4 patients with available data), but no patient had sepsis or shock. The main presenting clinical symptom was abdominal pain. The median duration of treatment was 20 days. No patient died, but 40% (2) had to be transitioned to hemodialysis after the episode of PD-associated peritonitis.

### 3.10. Cellulosimicrobium Species Endophthalmitis

The review revealed five patients with *Cellulosimicrobium* species endophthalmitis; their median age was 72 years (ranging from 28 to 78), and 60% (3 of them) were male. Among these five patients, 60% (3) had surgery during the previous three months, and 40% (2) had recent ocular trauma. The diagnosis was established by the culture of intraocular fluid. The infection was exogenous in all patients. Symptoms were only local. Despite antimicrobial treatment, vitrectomy was required in all cases, and the outcome was optimal in all of them, with only a relative reduction of visual acuity.

## 4. Discussion

The present review summarizes the characteristics of patients with *Cellulosimicrobium* species infections based on previously published data and provides information regarding epidemiology, microbiology, clinical characteristics, treatment, and outcome. The most common infection types were those of the bloodstream: IE, osteoarticular infections, PD-associated peritonitis, and endophthalmitis. The most commonly used antimicrobial therapies for treating these infections were vancomycin, cephalosporins, and the combination of trimethoprim and sulfamethoxazole. Mortality was notable, mainly associated with bacteremia and IE.

Few reports of *Cellulosimicrobium* species infections in humans exist in the literature so far. Thus, the epidemiological, microbiological, and clinical characteristics have not been adequately described. The present review has revealed 40 patients with adequate information regarding *Cellulosimicrobium* species infections. Most patients were male, and the median age was 52.5 years. Most studies were conducted in North America and Europe. However, this geographic distribution may be due to the presence of advanced diagnostic techniques, such as MALDI-TOF MS and 16s rRNA, used in these geographical areas for pathogens’ identification since such techniques are more commonly found in the Western world. Hence, the geographic distribution shown herein may not represent the true epidemiology of the pathogens since, in areas where such diagnostic techniques are unavailable, the pathogens may have probably been misidentified.

This review identified different predisposing factors for different types of *Cellulosimicrobium* species infections. Half of the patients with *Cellulosimicrobium* species bacteremia had a CVC at diagnosis. At the same time, antimicrobial therapy use during the previous three months was also common among these patients. Finally, about one out of four patients with bacteremia had a history of an active malignancy. These characteristics imply a mode of transmission that is mainly of healthcare-associated origin. Infections such as the respiratory tract, urinary tract, surgical sites, and bloodstream are a few common examples of healthcare-associated infections (HAIs). The most common pathogens associated with HAIs are *S. aureus*, Enterobacterales, *Pseudomonas aeruginosa*, *Acinetobacter baumannii*, *Enterococcus* species, and *Candida* species [43]. HAIs constitute a significant concern in everyday practice since they are potentially preventable causes of patient harm. Hence, implementing specific measures by healthcare practitioners, such as hand hygiene and appropriate procedures for inserting and maintaining CVCs and urinary tract catheters, can significantly reduce HAIs [44,45,46,47,48]. This implies that implementing an appropriate infection control process could reduce the likelihood of bacteremia caused by *Cellulosimicrobium* species, among other more frequent pathogens. However, even though these infections could be healthcare-associated, community-associated acquisition may also be frequent, at least in some patients. For example, species of the genus *Cellulosimicrobium* have been identified as members of the salivary microbiome [49]. The fact that patients with bacteremia by these pathogens were frequently diagnosed with active malignancy and were more commonly on chemotherapy and neutropenics could imply an increased likelihood for the development of infection among patients with significant immunosuppression. Indeed, immunosuppression is associated with an increased risk of infection [50], and patients with cancer have an increased likelihood of infections by common pathogens as well as by uncommon, less frequent ones [51]. The type of immunosuppression is the main predicament for the pathogen that causes infection in immunosuppressed individuals. Hence, in cases of defects in cell-mediated immunity, infections by *Nocardia*, *Cryptococcus*, *Pneumocystis jiroveci*, *Histoplasma*, or other endemic dimorphic fungi are more frequent [51]. In patients with neutropenia, as in the case of patients with bacteremia in the present review, *P. aeruginosa*, *Aspergillus*, *Mucor*, and other pathogens are more frequent [51].

The present study noted classical predisposing factors associated with IE in patients infected by *Cellulosimicrobium* species, which included prosthetic cardiac valves, previous episodes of IE, and recent cardiac surgery. These factors have been extensively described in the literature among patients with IE [52,53,54].

PD is a classical risk factor for peritonitis. In these patients, Gram-positive pathogens are more common, including staphylococci, both coagulase-negative and *S. aureus*, with methicillin resistance being a significant problem from a clinical perspective [55,56]. However, other pathogens such as Gram-negative bacteria, fungi, mycobacteria, or rare Gram-positive bacilli have also been described as causes of PD-associated peritonitis [57]. This underlines the need for clinicians caring for such patients to be aware of the likelihood of the identification of rare pathogens.

Finally, in patients with endophthalmitis, trauma or recent surgery were noted in the history of all patients, thus defining these infections as exogenous. Indeed, exogenous endophthalmitis is the most common type of endophthalmitis, with Gram-positive pathogens, mainly coagulase-negative staphylococci, being among the most commonly identified pathogens [58]. This may reflect the strong association of this condition with recent ophthalmological surgical procedures.

Identifying rare bacteria, such as *Cellulosimicrobium* species, could be difficult from a microbiological perspective. Indeed, in the present review, advanced molecular methods such as MALDI-TOF or 16s rRNA gene sequencing were commonly used for pathogen identification. These advanced techniques may be required for accurate pathogen identification in cases of rare bacteria but may be relatively unavailable by the majority of microbiological laboratories [59].

Given the rarity of *Cellulosimicrobium* species, there are no guidelines for treating infections caused by this microorganism. Thus, the antimicrobial resistance patterns of these species are of great interest. The majority of studies included herein provided some information about the antimicrobial susceptibility of these species. The available data showed high resistance to clindamycin, aminopenicillins, penicillin, cephalosporins, quinolones, and aminoglycosides. On the contrary, antimicrobial resistance to the combination of trimethoprim with sulfamethoxazole was minimal, and resistance to vancomycin was zero. Notably, the present review has revealed that *Cellulosimicrobium* species infections may frequently be healthcare-associated. This may be of clinical importance in terms of antimicrobial resistance since resistant pathogens prevail in the hospital environment, either by selection of resistant microorganisms after exposure to antimicrobials or by transfer of genes providing resistance from other microorganisms [60,61,62]. Thus, even though this microorganism’s resistance mechanisms have not been elucidated, and it is unknown whether mobile genetic elements could lead to acquired resistance from other multidrug-resistant hospital bacteria, the possibility of developing significant antimicrobial resistance may exist [63]. It is of interest that scarce literature suggests that specific isolates of unknown clinical significance may have multiple mechanisms of resistance involving multidrug efflux pumps, multidrug-resistant transporters, metallo-beta-lactamases, and even a vancomycin-resistant protein [64]. Hence, it sounds reasonable that the most frequently used antimicrobial agent in the studies included in this review was vancomycin. Based on these data, clinicians caring for patients with these infections could use vancomycin as an empirical treatment until antimicrobial susceptibility data are available to allow appropriate de-escalation.

In the present review, mortality was high in bacteremic patients, which could have been related to their active malignancy or the associated treatment [65]. Mortality was even higher in patients with IE and was higher compared to cases of IE by other microorganisms reported in the literature [52,53,54]. However, the small number of patients with IE in the present review may preclude the extraction of firm conclusions. Larger numbers of patients with IE should be studied to evaluate the outcome of IE by this pathogen more accurately.

The present review has some limitations. First, despite the thorough search methodology, some studies may have been missed due to the search strategy. Additionally, due to the rarity of these infections, only case reports and case series were included in the review. Finally, some information was missing in the original studies; thus, this review only presents and analyzes the available data from the included studies.

## 5. Conclusions

This study provides essential data about the epidemiology, clinical characteristics, microbiology, antimicrobial susceptibility, treatment, and outcomes of *Cellulosimicrobium* species infections. The most common infections were those of the bloodstream IE, osteoarticular infections, PD-associated peritonitis, and endophthalmitis. Among patients with bacteremia, active malignancy and the presence of CVC were common. Peritonitis only occurred in patients with PD, and endophthalmitis was exogenous due to recent surgery or trauma in all patients. Susceptibility to vancomycin and the combination of trimethoprim and sulfamethoxazole was very high, and vancomycin was the most commonly used antimicrobial for treatment. The infection’s outcome depended on its type, with mortality being high in patients with bacteremia and IEs and minimal in all other patients.

## Figures and Tables

**Table 1:** Characteristics of included studies reporting *Cellulosimicrobium* species infections in humans.

Author, Year	Number of Patients	Gender	Age (Years)	Type of Infection	Treatment (%)	Mortality (%)
Reller et al., 1975 [6]	1	Male	68	BSI, IE	Penicillin, aminopenicillin, macrolide, and TMP-SMX 1 (100) Surgical management 1 (100)	0 (0)
Cruickshank et al., 1979 [7]	1	Female	47	UTI	NR Surgical management 1 (100)	0 (0)
Kailath et al., 1988 [8]	1	Female	38	CNS infection	Penicillin, rifampicin 1 (100) Surgical management 1 (100)	0 (0)
LeProwse et al., 1989 [9]	1	Male	3	BSI	Aminoglycosie 1 (100) Catheter removal 1 (100)	0 (0)
Guss et al., 1989 [10]	1	Female	40	BSI	Vancomycin 1 (100)	0 (0)
Rihs et al., 1990 [11]	1	Male	70	Peritonitis	Vancomycin, aminoglycoside 1 (100) Catheter removal 1 (100)	0 (0)
Truant et al., 1992 [12]	1	Male	40	BSI, RTI	Clindamycin, quinolone, vancomycin, and cephalosporin 1 (100)	1 (100)
McDonald et al., 1994 [13]	1	Female	54	BSI, RTI	Vancomycin 1 (100)	0 (0)
Lair et al., 1996 [14]	1	Male	27	BSI	Aminoglycoside, carbapenem 1 (100) Catheter removal 1 (100)	0 (0)
Maguire et al., 1996 [15]	1	Female	49	BSI	Vancomycin 1 (100)	0 (0)
Harrington et al., 1996 [16]	1	Male	72	Bone and Joint	TMP-SMX, vancomycin 1 (100) Surgical management 1 (100)	0 (0)
Borra et al., 1996 [17]	1	Female	59	Peritonitis	Vancomycin, tetracycline 1 (100)	0 (0)
Ellerbroek et al., 1998 [18]	1	Female	53	BSI, IE	Clindamycin, TMP-SMX, rifampicin, aminoglycoside, macrolide, carbapenem, and aminopenicillin 1 (100) Catheter removal 1 (100)	1 (100)
Lujan-Zilbermann et al., 1999 [19]	1	Female	13	Peritonitis	Vancomycin 1 (100)	0 (0)
Urbina et al., 2003 [20]	1	Male	30	BSI, IE	Aminopenicillin/b-lactam inhibitor, cephalosporin 1 (100) Surgical management 1 (100)	0 (0)
Heym et al., 2005 [21]	1	Male	48	Tongue	Macrolide, penicillin 1 (100)	0 (0)
Rowlinson et al., 2006 [22]	1	Male	13	BSI	Cephalosporin, vancomycin, aminoglycoside, and rifampicin 1 (100)	0 (0)
Thomas et al., 2007 [23]	1	Male	76	Cholecystitis	NR Surgical management 1 (100)	0 (0)
Tucker et al., 2008 [24]	1	Male	5	Bone and Joint	TMP-SMX, rifampicin 1 (100) Surgical management 1 (100)	0 (0)
Lai et al., 2009 [25]	1	Female	78	BSI, IE, bone and joint	Penicillin, aminoglycoside, TMP-SMX 1 (100)	0 (0)
Casanova-Román et al., 2010 [26]	1	Male	0	BSI	Vancomycin 1 (100)	0 (0)
Akçakaya et al., 2011 [27]	3	2 females 1 male	72, 74, 78	3 Endophthalmitides	Cephalosporin, vancomycin 3 (100) Surgical management 3 (100)	0 (0)
Jaru-Ampornpan et al., 2011 [28]	1	Male	28	Endophthalmitis	Quinolone, vancomycin, cephalosporin, and clindamycin 1 (100) Surgical management 1 (100)	0 (0)
Petkar et al., 2011 [29]	1	Male	81	BSI, IE	Aminoglycoside, vancomycin 1 (100)	1 (100)
Castellanos et al., 2011 [30]	1	Male	62	Peritonitis	Vancomycin, quinolone, aminoglycoside, and TMP-SMX 1 (100) Catheter removal 1 (100)	0 (0)
Magro-Checa et al., 2011 [31]	1	Male	81	Bone and Joint	Rifampicin, linezolid 1 (100) Surgical management 1 (100)	0 (0)
Delport et al., 2014 [32]	1	Male	59	BSI	Piperacillin/tazobactam, vancomycin 1 (100)	1 (100)
Sug Kim et al., 2015 [33]	1	Female	50	Peritonitis	Aminoglycoside, vancomycin, and cephalosporin 1 (100) Catheter removal 1 (100)	0 (0)
Coletta-Griborio et al., 2017 [34]	1	Female	80	BSI	Vancomycin 1 (100) Catheter removal 1 (100)	1 (100)
Ponce-Alonso et al., 2017 [35]	1	Female	59	BSI	Vancomycin, carbapenem 1 (100) Catheter removal 1 (100)	0 (0)
Gonzales Zamora et al., 2018 [36]	1	Female	44	BSI	Vancomycin, carbapenem 1 (100) Catheter removal 1 (100)	1 (100)
Oikonomou et al., 2018 [37]	1	Male	50	BSI, bone and joint, skin	Cephalosporin, vancomycin, and TMP-SMX 1 (100) Surgical management 1 (100)	0 (0)
Monticelli et al., 2019 [38]	1	Female	66	BSI, IE	Linezolid, vancomycin, and carbapenem 1 (100)	1 (100)
Rivero et al., 2019 [39]	1	Female	82	BSI, IE	Aminopenicillin, vancomycin, and linezolid 1 (100)	1 (100)
Rohowetz et al., 2019 [40]	1	Male	49	Endophthalmitis	Vancomycin, cephalosporin 1 (100) Surgical management 1 (100)	0 (0)
Zhang et al., 2020 [5]	1	Female	52	BSI, IE	Vancomycin, rifampicin, and cephalosporin 1 (100)	1 (100)
Hernández Martínez et al., 2020 [41]	1	Male	78	BSI	Linezolid 1 (100)	0 (0)
Trindade Torres et al., 2024 [42]	1	Male	17	Bone and joint	TMP-SMXaminoglycoside 1 (100) Catheter removal 1 (100)	0 (0)

BSI: bloodstream infection; CNS: central nervous system; IE: infective endocarditis; TMP-SMX: trimethoprim-sulfamethoxazole; UTI: urinary tract infection.

**Table 2:** Characteristics of the different types of infections by *Cellulosimicrobium* species.

Characteristic *	All Patients (*n* = 40)	Bacteremia ** (*n* = 22)	Infective Endocarditis ** (*n* = 8)	Bone and Joint Infection (*n* = 6)	PD-Associated Peritonitis (*n* = 5)	Endophthalmitis (*n* = 5)
Age, median in years (IQR)	52.5 (38.5–72)	52.5 (37.5–70.5)	67 (52.3–80.3)	61 (14–78.8)	59 (31.5–66)	72 (38.5–76)
Male, *n* (%)	22 (55)	11 (50)	3 (37.5)	5 (83.3)	2 (40)	3 (60)
Post-surgery (within 3 months), *n* (%)	6 (15)	1 (4.5)	1 (12.5)	0 (0)	1 (20)	3 (60)
Post-cardiac surgery (within 3 months), *n* (%)	1 (2.5)	1 (4.5)	1 (12.5)	0 (0)	0 (0)	0 (0)
Previous antimicrobial therapy, *n* (%)	13/39 (33.3)	9/21 (42.9)	4 (50)	3 (50)	1 (20)	0 (0)
Active malignancy, *n* (%)	6 (15)	6 (27.3)	1 (12.5)	0 (0)	0 (0)	0 (0)
ESRD on PD, *n* (%)	5 (12.5)	0 (0)	0 (0)	0 (0)	5 (100)	0 (0)
ESRD on HD, *n* (%)	1 (2.5)	1 (4.5)	0 (0)	0 (0)	0 (0)	1 (100)
Polymicrobial infection, *n* (%)	5 (12.5)	2 (9.1)	0 (0)	1 (16.7)	1 (20)	0 (0)
Clinical characteristics						
Fever, *n* (%)	19/36 (52.8)	16/19 (84.2)	7 (87.5)	0 (0)	2/4 (50)	0 (0)
Sepsis, *n* (%)	11/38 (28.9)	11/21 (52.4)	4 (50)	1 (16.7)	0/4 (0)	0 (0)
Outcomes						
Overall mortality, *n* (%)	8 (40)	8 (36.4)	5 (62.5)	0 (0)	0 (0)	0 (0)
Infection-related mortality, *n* (%)	7 (17.5)	7 (31.8)	5 (62.5)	0 (0)	0 (0)	0 (0)

ESRD: end-stage renal disease; HD: hemodialysis; IQR: interquartile range; PD: peritoneal dialysis; * denominator is the total number of patients except if otherwise mentioned; ** cases of bacteremia include the eight cases of infective endocarditis.

**Table 3:** Characteristics of patients with bacteremia by *Cellulosimicrobium* species in regards to the outcome.

Characteristic *	Survived (*n* = 14)	Died (*n* = 8)
Age, median in years (IQR)	44.5 (23.5–61.3)	62.5 (52.3–80.8)
Male, *n* (%)	9 (64.3)	2 (25)
Post-surgery (within 3 months), *n* (%)	1 (7.1)	0 (0)
Post-cardiac surgery (within 3 months), *n* (%)	1 (7.1)	0 (0)
Previous antimicrobial therapy, *n* (%)	5/13 (38.5)	4 (50)
Active malignancy, *n* (%)	4 (28.6)	2 (25)
Neutropenia, *n* (%)	2/13 (15.4)	2 (25)
CVC, *n* (%)	7 (50)	4 (50)
Organ transplantation, *n* (%)	1 (7.1)	2 (25)
ESRD on HD, *n* (%)	0 (0)	1 (12.5)
Polymicrobial infection, *n* (%)	2 (14.3)	0 (0)
Clinical characteristics		
Fever, *n* (%)	10/12 (83.3)	6/7 (85.7)
Sepsis, *n* (%)	6 (42.9)	5/7 (71.4)
IE, *n* (%)	3 (21.4)	5 (62.5)

CVC: central venous catheter; ESRD: end-stage renal disease; IE: infective endocarditis; IQR: interquartile range; * denominator is the total number of patients except if otherwise mentioned.

## Data Availability

The data presented in this study are available on request from the corresponding author.
